# Working Memory and Hearing Aid Processing: Literature Findings, Future Directions, and Clinical Applications

**DOI:** 10.3389/fpsyg.2015.01894

**Published:** 2015-12-16

**Authors:** Pamela Souza, Kathryn Arehart, Tobias Neher

**Affiliations:** ^1^Communication Sciences and Disorders, Knowles Hearing Center, Northwestern University, EvanstonIL, USA; ^2^Speech, Language and Hearing Sciences, University of Colorado Boulder, BoulderCO, USA; ^3^Medizinische Physik and Cluster of Excellence Hearing4all, Carl von Ossietzky University of OldenburgOldenburg, Germany

**Keywords:** working memory capacity, reading span, hearing aid, wide-dynamic range compression, digital noise reduction, frequency compression

## Abstract

Working memory—the ability to process and store information—has been identified as an important aspect of speech perception in difficult listening environments. Working memory can be envisioned as a limited-capacity system which is engaged when an input signal cannot be readily matched to a stored representation or template. This “mismatch” is expected to occur more frequently when the signal is degraded. Because working memory capacity varies among individuals, those with smaller capacity are expected to demonstrate poorer speech understanding when speech is degraded, such as in background noise. However, it is less clear whether (and how) working memory should influence practical decisions, such as hearing treatment. Here, we consider the relationship between working memory capacity and response to specific hearing aid processing strategies. Three types of signal processing are considered, each of which will alter the acoustic signal: fast-acting wide-dynamic range compression, which smooths the amplitude envelope of the input signal; digital noise reduction, which may inadvertently remove speech signal components as it suppresses noise; and frequency compression, which alters the relationship between spectral peaks. For fast-acting wide-dynamic range compression, a growing body of data suggests that individuals with smaller working memory capacity may be more susceptible to such signal alterations, and may receive greater amplification benefit with “low alteration” processing. While the evidence for a relationship between wide-dynamic range compression and working memory appears robust, the effects of working memory on perceptual response to other forms of hearing aid signal processing are less clear cut. We conclude our review with a discussion of the opportunities (and challenges) in translating information on individual working memory into clinical treatment, including clinically feasible measures of working memory.

## The Role Of Working Memory In Speech Perception

Working memory—the ability to process and store information (Daneman and Carpenter, 1980; Miyake and Shah, 1999; Baddeley, 2000, 2012)—has been identified as an important aspect of speech perception in difficult listening environments. For instance, working memory is thought to play an active role in the maintenance of task-relevant information. Storage and processing of information are simultaneously carried out during a complex cognitive task. Those processes draw upon a common set of resources which can be allocated according to the various task demands. Because working memory can be envisioned as a limited-capacity system, there will be a trade-off: if more processing is required, less information can be stored, and vice versa. When working memory capacity is reached, both processes will be impaired.

A comprehensive description of the relationship between working memory and speech understanding is contained in the Ease of Language Understanding (ELU) model developed by Rönnberg et al. (2008, 2013). Briefly, the ELU model views language input as containing phonological, syntactic, prosodic, and semantic information. When the language input can be matched unambiguously to a phonological representation stored in long-term memory, lexical retrieval proceeds in an implicit (and relatively effortless) way. However, when the phonological representation is not readily matched to the phonological representation (because the incoming information is degraded in some way), working memory is explicitly deployed to reconcile a match. To reconcile a match, the listener may need to utilize semantic information, make inferences, or inhibit irrelevant information to assign meaning to the input. We can think of working memory being engaged to a greater extent when the speech signal is ambiguous or distorted; and engaged to a lesser extent when the speech signal is audible and undistorted. Following from that model, it seems reasonable to expect stronger associations between working memory capacity and speech recognition when speech is acoustically degraded and weaker associations when speech is audible and clear.

A number of empirical studies have supported this view, showing working memory capacity to be more strongly related to speech in noise than to speech in quiet (see Akeroyd, 2008; Besser et al., 2013 for reviews). This relationship has led to calls for including measures of working memory in diagnostic protocols (Weinstein, 2015), or in treatment planning (Remensnyder, 2012). Individuals who present with a range of communication difficulties will likely benefit from an understanding of the cognitive (and sensory) factors that influence their communication abilities. However, it is less clear how working memory should be applied to practical decisions, including the selection and fitting of hearing aids. The current paper seeks to address this issue.

## Measuring Working Memory

Working memory capacity is usually measured with complex span tests which require the participant to manipulate and recall information. For example, the participant may be asked to recall a list of digits or letters in reverse serial order, to solve problems, or to make a judgment about items prompted for recall. Most relevant to the current review are tests of verbal working memory, particularly the reading span test (Daneman and Carpenter, 1980; Baddeley et al., 1985). In a typical reading span paradigm, participants read a set of sentences and make a semantic judgment about each sentence (thereby engaging processing). After a block of sentences, participants are asked to recall as many test items as possible. The participant may be asked to recall the items in the same order as they were presented (serial recall) or allowed to recall the items in any order (free recall). The number of items recalled is used as a metric of working memory capacity. However, when interpreting working memory capacity, we must remember that working memory is, essentially, a composite ability. Reading span tests draw on a number of abilities including reading speed, phonological processing, speed of lexical processing, and executive functioning (Souza and Arehart, 2015; Souza et al., 2015). Those abilities may govern the reading span test’s predictive power.

Many studies have documented that working memory capacity varies among individuals (see Akeroyd, 2008 for review). For the majority of studies summarized below, the reading span test was used to measure working memory capacity. Where available, participants’ reading span scores are provided (**Tables 1**–**3**). For the most common administration and scoring methods, reading span scores for older adults (>60 years) are typically distributed with a mean of about 35–40% and a standard deviation of about 10%. Scores for younger adults (<30 years) are higher, but still show considerably variability among individuals (e.g., Füllgrabe et al., 2015; Souza and Arehart, 2015).

**Table 1:** Summary of studies which related working memory capacity (via reading span) to fast-acting wide-dynamic range compression (WDRC).

Authors (year)	Number of participants	Participant mean age in years (SD or age range)	Hearing aid processing	Reading span mean score in percent correct (SD or score range)	Recall	Significant hearing aid processing – working memory relationship?
Foo et al., 2007	32	70 (*SD* = 8)	Fast-acting WDRC	44% (*SD* = 11%)	Free	Yes
Ohlenforst et al., 2015	26	74 (range 61–92)	Fast-acting WDRC	36% (*SD* = 11%)	Free	Yes
Davies-Venn and Souza, 2014	28	65 (range 21–89)	Fast-acting WDRC	79%^∗^ (*SD* = 14%)	Free	Yes
Souza and Sirow, 2014	27	82 (range 62–100)	Fast-acting WDRC	34% (range 17–50)	Free	Yes


*^∗^Administered with participant-controlled timing (Conway et al., 2005).*

**Table 2:** Summary of studies which related working memory capacity (via reading span) to digital noise reduction.

Authors (year)	Number of participants	Participant mean age in years (SD or age range)	Hearing aid processing	Reading span mean score in percent correct (SD or score range)	Recall	Significant hearing aid processing – working memory relationship?
Desjardins and Doherty, 2014	12	66 (range 50–74)	Modulation-based NR	49% (*SD* = 10%)	Free	No
Ng et al., 2013	26	59 (range 32–65)	Ideal binary mask-based NR	43% (*SD* = 14%)	Serial	Yes
Ng et al., 2015	26	62 (range 56–65)	Ideal binary mask-based NR	42% (*SD* = 13%)	Serial	Yes
Arehart et al., 2015	31	70 (range 51–89)	Ideal binary mask-based NR	39% (range 13–63)	Free	Yes
Neher et al., 2014b	40	75 (range 60–84)	Binaural coherence-based NR	36% (range 19–56)	Free	Yes, for preference
Neher et al., 2014a	40	72 (range 60–82)	Binaural coherence-based NR	38% (range 19–57)	Free	No
Neher, 2014	60	72 (range 60–82)	Binaural coherence-based NR	38% (range 19–57)	Free	No


**Table 3:** Summary of studies which related working memory capacity (via reading span) to frequency compression.

Authors (year)	Number of participants	Participant mean age in years (SD or age range)	Hearing aid processing	Reading span mean score in percent correct (SD or score range)	Recall	Significant hearing aid processing – working memory relationship?
Ellis and Munro, 2015	12	76 (range 65–84)	Frequency compression	29% (*SD* = 11%)	Free	No
Arehart et al., 2013	26	72 (range 62–92)	Frequency compression	40% (*SD* = 12%)	Free	Yes
Souza et al., 2015	29	74 (range 49–89)	Frequency compression and fast-acting WDRC	38% (range 15–54)	Free	Yes


There are several reasons why signals transduced by hearing aids might interact with working memory. Although they are designed to improve audibility (and therefore speech perception), hearing aids, by their nature, alter the input signal. In contrast to linear hearing aids that merely provided overall gain and frequency shaping and as such minimally altered the input signal, modern digital hearing aids aim to enhance speech, suppress noise, eliminate acoustic feedback, and maintain comfortable loudness. To accomplish these goals, digital filtering and manipulation are applied, which can considerably alter the input signal (Kates, 2010). The following sections consider the acoustic effects of three common hearing aid processing strategies: fast-acting wide-dynamic range compression; digital noise reduction (NR); and frequency compression (FC). Each is related to empirical data and then also considered in the context of the ELU model.

## Fast-Acting Wide-Dynamic Range Compression

The purpose of wide-dynamic range compression (WDRC) is to improve audibility while maintaining loudness comfort. That goal is achieved by applying gain as a function of intensity, with lower gain applied to higher input levels. At a group level, WDRC has been shown to provide equivalent speech recognition to linear amplification at conversational levels, and improved audibility and loudness comfort for low- or high-intensity speech (Larson et al., 2000; Souza, 2002). To understand the relationship between WDRC and working memory, the next section describes some details of WDRC processing.

### Fast-Acting Wide-Dynamic Range Compression: Processing Principles

In a typical WDRC implementation, excessive input levels are managed by a front-end limiter. Next, the signal is filtered according to the number of compression channels (2–30 channels, depending on the hearing aid). The input intensity within each channel is monitored and gain is adjusted for inputs above a compression threshold (typically 40–50 dB SPL). In clinical fittings, compression ratios^1^ typically vary between 1:1 and 3:1. To avoid loudness discomfort, signals exceeding a given (“compression limiting”) threshold (typically 80–100 dB SPL) are subject to more extreme gain reduction (and higher compression ratios). The compressor gain function is determined by the total input signal, including the target speech and any background noise present in the environment.

Gain is adjusted dynamically as the input level changes. An important characteristic is the speed of the compressor, indicated by the attack and release times^2^, which together determine the compression speed. Although compression speed varies along a continuum, compression systems are often classified as fast- or slow-acting, where release times of less than 200 ms indicate a fast-acting WDRC system. In fast-acting WDRC systems, audibility may be high, but the amplitude envelope of the signal may be substantially altered relative to its natural amplitude pattern (Jenstad and Souza, 2005; Stone and Moore, 2007). Slow-acting compression systems adhere more closely to the natural amplitude envelope, but at the expense of improved consonant audibility. Slow-acting compression is sometimes marketed as providing improved sound quality, whereas fast-acting compression is marketed as more dynamic and able to respond more aggressively to changing inputs.

Decades of research have failed to reach consensus as to the “optimal” compression speed for improved speech recognition. Some studies showed better (group) performance with fast-acting WDRC, others with slow-acting WDRC (Souza, 2002). A feasible explanation for the conflicting evidence is that fast WDRC is the best option only for *some* listeners, and slow WDRC is the best option for others. In other words, there may be a trade-off between improved audibility and susceptibility to distortion of the amplitude patterns of the signal.

Recall that the ELU model proposes that working memory will be explicitly engaged (and working memory capacity will play a larger role) in cases where the phonological input cannot be immediately matched to its phonological representation in long-term memory. Because fast WDRC can result in greater alteration of the signal, it has been proposed that some WDRC parameters may increase the chance of match failure between the phonological input and the phonological representation in long-term memory. If that situation occurs, we expect to find a relationship between working memory capacity and understanding of speech amplified by fast-acting WDRC. The next sections review a series of studies that evaluated this relationship.

### Studies of WDRC Speed and Working Memory Capacity: Empirical Findings

In an influential study, Gatehouse et al. (2003, 2006a,b) explored how individual abilities modified the benefits of hearing aid signal processing. The authors were interested in a variety of predictors, including pure-tone thresholds, dynamic range, temporal, and spectral resolution, cognitive abilities, and the variability of sound levels in the listener’s daily listening environment. Data were obtained from experienced hearing-aid wearers who undertook a double-blind trial of amplification strategies which varied in compression speed. The cognitive tests consisted of letter- and digit-monitoring tests. Although not described as working memory tests, the cognitive tests required both processing and storage. Speech recognition was measured in a closed-set speech test. Cognitive ability was related to both reported and measured intelligibility such that listeners with higher cognitive scores also had higher intelligibility scores, but only for the hearing aid processing conditions which employed fast compression. Data were interpreted to suggest that individuals with greater capacity to store and process information would benefit to a greater extent from fast compression.

Lunner and Sundewall-Thorén (2007) replicated the Gatehouse work in a group of 23 experienced hearing-aid wearers but with an adaptive sentence-in-noise test, where background noises were speech-spectrum noise or two-talker modulated noise (Dreschler et al., 2001). As in the Gatehouse work, fast- and slow-acting WDRC were implemented in wearable hearing aids and participants used the aids for a period of acclimatization prior to testing. Consistent with Gatehouse et al. (2006b) low cognitive scores on a letter-monitoring test were associated with poorer performance with fast-acting WDRC. However, that relationship also depended on the type of noise. For example, for sentences in speech-spectrum noise amplified with slow compression, pure-tone average explained nearly 30% of variance in speech scores, with cognitive ability accounting for only 5%. For sentences in modulated noise amplified with fast compression, pure-tone average explained less than 5% of variance, and cognitive ability accounted for nearly 40%. These data patterns can be interpreted to suggest that as signal complexity increases (either through the presence of noise modulation, or application of fast WDRC), the role of cognition increases and the role of audibility decreases.

Foo et al. (2007) evaluated working memory and the effect of compression speed in experienced hearing-aid wearers. All participants completed the reading span test. Two different sentence recognition tests were completed: the Hagerman sentences (Hagerman and Kinnefors, 1995) in one-talker modulated noise and in unmodulated noise; and the HINT sentences (Nilsson et al., 1994) in two-talker ICRA noise (Dreschler et al., 2001) and in unmodulated noise. The HINT sentences have higher predictability than the Hagerman sentences. Each speech-in-noise test was performed with fast and slow WDRC. For the Hagerman sentences, there was an interaction between compression speed and reading span score, such that listeners with low working memory performed more poorly with fast WDRC. For the HINT sentences, there was no interaction between compression speed and reading span score. There was an interaction between compression speed and a second cognitive test (letter monitoring). Listeners who scored more poorly on the letter monitoring test performed more poorly with slow WDRC. However, the study authors also speculated that because letter monitoring is a serial task, it may capture different (and less relevant) aspects of cognition than the dual store-and-process tasks required in the reading span test and during speech recognition.

Ohlenforst et al. (2015) delved further into this relationship, focusing on the modulation characteristics of the background noise. Working memory capacity was assessed with the reading span test. Older participants were grouped by high or low working memory according to their reading span scores. Speech intelligibility was measured for low-context sentences presented in background noise, where the noise varied in the extent of modulation (1-, 2-, and 6-talker ICRA noise). Fast- or slow-acting WDRC was created in a laboratory simulation. As in Gatehouse et al. (2006b) and in Lunner and Sundewall-Thorén (2007), Ohlenforst et al. (2015) demonstrated a relationship between cognitive ability and compression speed. Listeners with high working memory demonstrated higher speech recognition scores when fast compression was applied than when slow compression was applied. In contrast, the low working memory group performed better with slow compression compared to fast compression. The magnitude of the score difference between compression speeds depended on the number of talkers in the background noise, with the largest differences for the highly modulated noises. However, noise modulation did not interact with working memory.

In a more clinical implementation, Souza and Sirow (2014) measured working memory capacity (via the reading span test) in older adults seen for hearing evaluations in an audiology clinic. Speech recognition was measured for sentences in noise (four-talker babble) using hearing aids with a range of compression speeds. All aids were adjusted to the same prescriptive target with an omnidirectional microphone, but had different numbers of compression channels and digital NR and feedback settings. Encouragingly, the relationships between working memory and compression speed followed those shown in more controlled, laboratory-based studies. The relative influence of working memory, amount of hearing loss, and age to speech recognition depended on the speed of the compression processor. For slow-acting compression, speech recognition was affected by age and amount of hearing loss but not related to working memory capacity. For fast-acting compression, working memory capacity accounted for 30% of the variance in speech understanding.

Although most studies which examined the relationships between speech understanding and working memory did so for speech in noise, working memory capacity may also help listeners with resolving a mismatch for specific phonemes in quiet. Davies-Venn and Souza (2014) processed vowel-consonant-vowel syllables with fast-acting WDRC. A range of compression ratios and release times were used to create stimulus sets with different degrees of acoustic alteration (and, presumably, a greater or lesser chance of a missed lexical match). The participants were adults with hearing losses ranging from mild to severe. Working memory capacity was measured using the reading span test with participant-controlled timing, which resulted in a similar variance but higher overall scores. The authors also considered signal audibility and spectral resolution, hypothesizing that listeners with poor spectral resolution would be most susceptible to the smoothed amplitude contours from the WDRC processing. Working memory, signal audibility and spectral resolution were all related to the effects of WDRC processing. The predictive value of working memory was strongest for the listeners with more hearing loss. That finding is consistent with the ELU model, as those listeners would be expected to experience the greatest “mismatch” due to their more severe hearing loss.

In summary, there is growing consensus that the response to specific compression parameters may be affected by individual working memory capacity. A number of studies (**Table 1**) have shown that listeners with smaller working memory capacity have more difficulty understanding speech processed by fast WDRC than by slow WDRC. However, that conclusion must be qualified, as it may apply only to populations, materials and hearing aid fittings that have been tested. In the next sections, we consider some variables that may modify the strength of the working memory-compression speed relationship.

#### Does Previous Exposure to Fast-Acting WDRC Matter?

The existing data support an association between smaller working memory capacity and poorer response to fast WDRC. In some of those studies, relationships between working memory and WDRC speed were noted as the listener was presented with a previously unfamiliar type of WDRC processing (e.g., Foo et al., 2007; Souza and Sirow, 2014; Ohlenforst et al., 2015). We might reasonably ask: will the relationship persist after “getting used to” new processing? Rudner et al. (2009) have argued that susceptibility to signal alteration (as with listeners with low working memory presented with fast WDRC) would be greatest in cases where the device processing presents a “mismatch”. Because slow WDRC more closely preserves natural speech amplitude patterns, we expect fast-acting WDRC to cause the greatest mismatch. After wearing the new processing for a period of time, the listener might “relearn” the new acoustic representations and store those representations in long-term memory, diminishing the mismatch problem and dissolving the working memory-compression speed relationship. Rudner et al. (2009) presented data in support of this idea, in that working memory and sentence-in-noise understanding were more likely to be related when speech was amplified with a compression speed that was unfamiliar to the listener; and less likely to be related when speech was processed with a WDRC speed familiar to the listener. However, a requirement that the processing be unfamiliar to generate a mismatch (and hence a working memory relationship) cannot be universally true. At least, the demonstration of a working memory-compression speed relationship was maintained even after multi-week experience with the specific processing under study (Gatehouse et al., 2003, 2006a,b; Lunner and Sundewall-Thorén, 2007; Rudner et al., 2011). It is possible that longer exposure (months or years) would result in different relationships. Although long-term acclimatization may or may not alter the role of working memory, most authors have assumed that experience counts, and that the most empirically valid conclusions can be drawn after acclimatization to processing.

#### Does the Speech Material Matter?

If we consider the strength of the working memory capacity-by-compression speed relationship in the context of the ELU model, we expect a stronger relationship when there is a greater chance of match failure. This has already been shown by the fact that working memory capacity tends to predict speech in noise performance when signal components are likely to be masked or ambiguous, but not speech understanding in quiet where signal components are audible and clear. In that theme, it may be of value to consider the relationship between working memory and compression speed relative to the acoustic properties of the speech undergoing compression. Although multichannel compression can also introduce spectral changes, a dominant feature is smoothing of the speech envelope. Presumably, for speech materials where envelope cues are relatively more important, the consequences of compression speed will be larger. Several authors have noted that envelope cues are of relatively greater importance for sentence perception compared to short-duration (syllable or bisyllabic word) perception (e.g., Van Tasell and Trine, 1996; Fogerty and Humes, 2012). Accordingly, the consequences of working memory may be more strongly demonstrated for compressed sentence- or narrative-length speech materials.

That idea could also be carried forward to the linguistic content of the speech. Highly predictable speech could be regarded as an “easy match,” with fewer ambiguities and therefore a lesser role of working memory. Less predictable speech might require a heavier processing load, more storage, and more rapid evaluation for meaning. In support of this idea, Cox and Xu (2010) assessed cognitive abilities, speech recognition and user preferences for 24 experienced hearing-aid wearers. Speech recognition tests included high-predictability sentences in four-talker babble, and a closed-set monosyllabic word test in modulated and unmodulated noise (similar to the closed-set test employed by Gatehouse et al. (2006a,b)). As had been the case in previous studies, speech recognition was compared for fast-acting WDRC and slow-acting WDRC. Cox and Xu’s (2010) paradigm differed somewhat from previous work, in that they deliberately selected listeners with very low or very high cognitive scores for comparison (*n* = 8 participants per group). Cognitive ability was quantified with three test scores (including the visual letter monitoring test employed by Gatehouse et al. (2006b), but not including a reading span test) collapsed into a composite score. Two interesting findings emerged. First, Cox and Xu’s (2010) sentence results did not reproduce the working memory-by-compression speed effect reviewed above. In fact, when only the visual letter monitoring task was used as a predictor (as by Lunner and Sundewall-Thorén, 2007), listeners with low cognitive scores performed worse (not better) with slow WDRC compared to fast WDRC. Cox and Xu (2010) concurred with previous authors that working memory contributed to the effect of WDRC processing. Unlike previous authors, they highlighted a potential effect of the speech materials: that for high-predictability materials, listeners with smaller working memory capacity might require fast WDRC (and its accompanying audibility improvements) for best performance. That suggestion returns to a point raised earlier in this review: that the choice of compression speed may create an audibility-by-distortion balance. In that theme, the net benefit depends on the listener (e.g., severity of hearing loss, susceptibility to signal distortion) and, perhaps, on the environment and/or the speech material. Given the small size of the comparison groups, replication of the Cox and Xu work with a larger sample would be valuable in untangling this issue.

### Summary

Among the studies described above, some general conclusions can be drawn. The effect of compression release time seems to be less important for listeners with larger working memory capacity. Those individuals perform better overall than listeners of similar age and audiometric status but with smaller working memory capacity. They also show minimal effects of varying compression release time. When release time makes a difference to individuals with larger working memory capacity, they perform better with fast compression. Similar to previous authors, we interpret these data to suggest that participants with larger working memory capacity have better abilities to store and process information simultaneously, which allows them to cope with distortion introduced by the fast compressor. One positive effect of fast compression is the potential to amplify brief speech segments in the target speech signal. Individuals with larger working memory capacity seemed to have the ability to better utilize the amplified information and, perhaps, to distinguish between helpful information and phonetic artifacts created by the compressor.

The effect of compression release time seems to be most consequential for listeners with smaller working memory capacity. Across most studies, these individuals show greater benefit from the less-distorting slow compressor. Presumably, when confronted with an acoustically altered signal, those individuals are less able to deploy cognitive resources to achieve a lexical match, preventing them from obtaining full benefit from the greater signal audibility. That pattern may also depend on the speech materials, particularly predictable vs. ambiguous syntax. The acoustic environment may also play an important role. Several studies have shown that the working memory by compressor speed interaction is largest when modulated background noise is present. Finally, it is possible that these effects will be moderated by prior long-term use of fast compression.

## Digital Noise Reduction

Where the input to the hearing aid is a mixed speech and noise signal, digital NR aims to identify and suppress noise components while preserving the speech components. When the background noise is other speech, digital NR is unlikely to result in improved speech perception. However, it may have other benefits, including greater sound comfort (Bentler, 2005; Bentler et al., 2008; McCreery et al., 2012). To understand the relationship between NR and working memory, the next section describes some details of digital NR.

### Digital Noise Reduction: Processing Principles

The main purpose of digital NR is to reduce the adverse effects of background noise on speech. This is achieved by means of an algorithm that estimates the presence (or absence) of speech in a noisy input signal. Once a signal segment has been classified as being noise- or speech-dominated, amplification can be applied to that segment in order to attenuate the noise and/or enhance the speech (e.g., Kates, 2008).

Various approaches have been developed for detecting speech in a noisy input signal. As a consequence, NR systems can differ widely in terms of their design principles and hence their efficacy under different acoustical conditions. In general, NR systems have several common features: they estimate the presence of speech based on one or more signal features; they perform the processing in a number of frequency bands; and they involve a trade-off between the amount of noise suppression achieved and the amount of artifacts introduced concurrently.

In the following, we will briefly describe three types of NR processing that have recently been tested in studies concerned with the influence of working memory capacity on NR outcome: (1) modulation-based NR processing, (2) binary mask-based NR processing, and (3) binaural coherence-based NR processing.

#### Modulation-Based Noise Reduction Processing

A characteristic feature of human speech is that – in contrast to many noise signals – it contains strong amplitude modulations, especially in the 3–4 Hz range (e.g., Drullman et al., 1994). Therefore, one approach to the design of a NR system is to use modulation depth as a criterion for the detection of speech. Signal segments containing strong modulations are classified as speech and are preserved, whereas signal segments with little modulation are classified as noise and are attenuated (e.g., Holube et al., 1999). The overall effect of the processing varies with the time scale over which the estimation and attenuation occur, and also with the strength of the attenuation. Because speech and noise signals vary over time, performing the processing on shorter time segments allows the algorithm to better track these variations. In principle, the classifications will reflect the actual short-time properties of the input signal. Nevertheless, misclassifications may also occur, especially for shorter time scales (where the estimates will be based on fewer observations). Increasing the strength of attenuation can lead to better noise suppression for signal segments that are accurately classified as being noise-dominated. For misclassifications, however, this will result in greater attenuation and thus distortion of the wanted signal. Thus, in the parameterization of a NR algorithm a trade-off exists between noise suppression and speech distortion.

#### Binary Mask-Based Noise Reduction Processing

An alternative (and more recent) approach to noise suppression is the use of so-called binary masks (e.g., Wang, 2008; Wang et al., 2009). Essentially, a binary mask is a matrix of zeros and ones that index the presence or absence of speech information in a noisy signal mixture as a function of time and frequency. Each zero or one corresponds to a given time-frequency unit. A one denotes a speech-dominated unit and a zero denotes a noise-dominated unit. Whether a given unit is assigned a zero or a one depends on the signal-to-noise ratio (SNR) of that unit (the ‘local SNR’). If the SNR exceeds a certain threshold (e.g., 0 dB) the unit is assigned a one; otherwise it is assigned a zero. The resultant pattern of zeros and ones is then used as a time- and frequency-dependent gain function that is applied to the original signal mixture, attenuating the noisy time-frequency units.

A notable problem with the binary mask-based approach is how to estimate the local SNRs accurately. In earlier studies, *ideal* binary masks were used to investigate the perceptual consequences of this type of processing (e.g., Anzalone et al., 2006; Wang et al., 2009). Ideal binary masks have *a priori* knowledge of the local SNRs (i.e., they do not need to estimate them). In a wearable hearing aid with no opportunity for prior knowledge of the signal, the mask must make do with non-ideal speech and noise detectors. More recently, some researchers have included a more realistic form of binary mask-based NR processing in their studies (Ng et al., 2013, 2015). With that type of processing, the local SNRs are estimated based on the output signals of two directional microphones, one facing forward in the direction of the target speech (thereby providing a relatively ‘clean’ speech signal) and the other one facing backward in the direction of the interfering signals (thereby providing a relatively ‘clean’ noise signal; cf., Boldt et al., 2008).

Binary mask-based NR processing is subject to the constraints concerning time scales and attenuation strengths outlined above. In addition, an SNR threshold for distinguishing between speech- and noise-dominated units has to be chosen. Binary mask-based NR processing can therefore also produce distortions that offset the benefit from the noise suppression, especially for realistic binary mask-based applications where speech and noise signals have to be estimated.

#### Binaural Coherence-Based Noise Reduction Processing

A third approach to the estimation of useful and detrimental acoustic information relies on the across-ear comparison of noisy input signals. This type of algorithm exploits the interaural similarity or binaural coherence as a decision metric for distinguishing between target signals and interferers (e.g., Grimm et al., 2009). As such, it requires the exchange of information across hearing instruments (e.g., using a wireless link). An implicit assumption made in the design of this algorithm is that incoherent signal components constitute detrimental information for the user (because they typically are due to strong reflections or diffuse background noise) and can be attenuated. First, the binaural coherence of the ear input signals is estimated as a function of time and frequency. The estimates produced in this manner can take on values between 0 and 1. A value of 0 corresponds to fully incoherent (or diffuse) sound, while a value of 1 corresponds to fully coherent (or directional) sound. Because of diffraction effects around the head, the coherence is always high at low frequencies. At frequencies above about 1 kHz, the coherence is low for diffuse and reverberant signal components, but high for the direct sound from nearby directional sources (e.g., talkers). Due to the spectro-temporal fluctuations contained in speech, the ratio between incoherent and coherent signal components may vary across time and frequency. By applying appropriate time- and frequency-dependent gains to the noisy input signals, this ratio can be improved. Once again, greater noise suppression comes at the expense of greater distortion of presumably useful signal components such as speech signals from nearby talkers (cf., Neher, 2014).

### Digital Noise Reduction and Working Memory Capacity: Empirical Findings

Recently, a number of studies have also investigated the relationship between working memory capacity (as indexed by the reading span test) and NR outcome, which are summarized below.

#### Modulation-Based Noise Reduction Processing

Desjardins and Doherty (2014) conducted a study to investigate listening effort with a modulation-based NR algorithm implemented in wearable (commercial) behind-the-ear hearing aids. Twelve mostly elderly hearing aid users participated. Amplification was prescribed in accordance with the DSL fitting rule (Scollie et al., 2005). Outcome was assessed using a dual-task paradigm combining speech understanding with a visual tracking task. A correlation analysis was conducted to explore the influence of working memory as well as performance on a measure of “processing speed” on visual tracking performance (i.e., the authors’ measure of listening effort). No correlations were observed. However, there was a trend for participants with faster processing speed to perform better on the visual tracking task when NR was engaged.

#### Binary Mask-Based Noise Reduction Processing

With respect to binary mask-based NR processing, Ng et al. (2013) conducted a study where they tested both ideal and non-ideal versions of this algorithm. Stimulus presentation was via insert earphones and included proprietary linear amplification. Participants were 26 mostly middle-aged hearing aid users. Outcome was assessed using a paradigm that required participants to identify the final words of a set of sentence-in-noise stimuli and then recall them afterwards. Data analyses revealed a main effect of working memory capacity on recall, with better memory being related to longer working memory capacity. Furthermore, an interaction between working memory capacity and non-ideal NR processing was observed. That is, participants with larger working memory capacity (measured using a reading span test) recalled more words from a speech recognition task than participants with smaller working memory capacity as a result of NR processing.

In a follow-up experiment based on essentially the same setup, Ng et al. (2015) tested the non-ideal algorithm further. A group of mostly older hearing aid users participated. Again, outcome was assessed in terms of sentence-final word identification and recall. Data analyses confirmed the previously observed effect of reading span on recall. Also, a two-way interaction between working memory capacity, NR processing, and serial word position was observed. That is, participants with smaller working memory capacity achieved better memory performance due to NR processing for the final word position only, whereas participants with larger working memory capacity achieved better memory performance irrespective of sentence word position.

Arehart et al. (2015) tested ideal binary mask-based NR processing as well as several non-ideal versions obtained through systematic manipulation of two algorithmic parameters (i.e., error rate and attenuation strength). Participants were mostly elderly hearing-impaired listeners, including 14 hearing aid users. Stimulus presentation was via headphones with linear amplification prescribed according to the NAL-R (Byrne and Dillon, 1986) fitting rule. Both speech understanding and speech quality were assessed. Data analysis revealed that working memory capacity was a significant predictor of overall intelligibility, but did not interact with the level of signal distortion in explaining performance.

#### Binaural Coherence-Based Noise Reduction Processing

Concerning binaural coherence-based NR processing, Neher et al. (2014b) carried out a headphone experiment with a hearing aid simulator that, in addition to NR processing, provided NAL-R amplification. Participants were elderly hearing aid users. A dual-task paradigm combining speech understanding with visual response time was used to assess performance. Pairwise preference comparisons were also collected. Regarding speech understanding, data analyses revealed a main effect of working memory capacity, but no interaction with NR processing. Regarding visual response times, no influence of working memory capacity was found. Regarding overall preference, participants with smaller working memory capacity preferred stronger NR processing than participants with larger working memory capacity.

Using a similar setup and almost the same group of participants, Neher et al. (2014a) tested a number of additional binaural coherence-based NR conditions. Outcome measures included the dual-task paradigm used previously as well as subjective ratings of listening effort and overall preference. This time, working memory capacity was unrelated to speech understanding and did not interact with NR processing either.

Again using a similar setup but this time a group of completely different elderly hearing aid users, Neher (2014) assessed speech understanding and also collected pairwise preference comparisons at a number of fixed SNRs. Participants’ performance on a visual measure of “executive control” (designed to tap into cognitive functions such as working memory, mental flexibility, and selective attention) was also considered. Regarding speech understanding, larger working memory capacity was once again associated with better performance. Furthermore, working memory capacity interacted with NR processing at 0 (but not -4) dB SNR. That is, while participants with larger working memory capacity showed a (statistically significant) performance decrement of a few percentage points due to (strong but not moderate) NR processing, participants with smaller working memory capacity did not. Regarding overall preference, no effects of working memory capacity were found. However, NR processing interacted with executive control at 0 and 4 (but not -4) dB SNR, i.e., participants with poorer executive control preferred stronger NR than participants with better executive control.

### Summary

Out of the seven studies on DNR and working memory summarized above (**Table 2**), five observed a general influence of working memory capacity on speech-in-noise performance (assessed in terms of speech intelligibility or memory performance), thereby confirming the positive relationship between working memory capacity and basic speech understanding abilities reported previously (e.g., Akeroyd, 2008). In contrast, only three studies observed an interaction between working memory and NR processing, two of which assessed memory performance and the other one speech intelligibility. Furthermore, across these three studies working memory capacity was inconsistently related to NR outcome. That is, while the two studies on memory performance found longer working memory to be associated with (larger) benefit from (binary mask-based) NR processing, the study on speech intelligibility found larger working memory capacity to be associated with *dis*benefit from strong (but not moderate, binaural coherence-based) NR processing. Although a fourth study indicated a relation between smaller working memory capacity and preference for (stronger binaural coherence-based) NR processing, subsequent studies failed to replicate this. However, one study found a corresponding relation between performance on a measure of executive control and preference for (binaural coherence-based) NR processing.

Because of these divergent findings, it is not straightforward to reconcile them with the ELU model. As pointed out above, the ELU model postulates a larger influence of working memory capacity when the phonological input cannot be immediately matched to its phonological representation in long-term memory. If one assumes that stronger NR processing results in greater alteration of the input signal, one would expect a relationship between larger working memory capacity and better understanding (or recall) of noise-reduced speech, but this was generally not the case. A possible reason for this could be that stronger NR processing may be having two concurrent effects: improving audibility of the speech signal (by suppressing more noise) and introducing more distortion than less aggressive processing. Perhaps the net effect of these competing factors contributes to the weak relationship between reading span and NR. It could also be that in some studies the effects of NR processing were kept constant across participants, while in others they were not (e.g., if the effects of NR co-varied with the prescribed amplification, as may be the case in commercial devices).

In summary, although working memory capacity is generally associated with speech perception, it seems to barely interact with NR outcome. In this context, however, it should be noted that the experimental conditions (e.g., the types of algorithm or outcome measures used) differed rather widely across studies (probably much more so than across the studies on WDRC), making a direct comparison difficult.

## Frequency Compression

The goal of FC is to increase the audibility of higher-frequency phonemes (where a patient typically has significant hearing loss) by restricting them to lower frequency regions (where the patient has better thresholds). The following section describes some details of this processing.

### Frequency Compression: Processing Principles

Several different implementations of FC have been used in simulated and commercial hearing aids (see Alexander, 2013 for a review). In one approach, the input speech signal is represented as a sum of sinewaves with characteristic frequencies, amplitudes and phases. When the speech signal is compressed, the modeled sine waves in the higher-frequency portions of the signal are reproduced at lower frequencies. The shifting of the higher-frequency energy to lower-frequency regions alters the fidelity of the incoming speech signal. Frequency compression may modify the signal envelope by changing the modulation structure within auditory bands, and will also reduce frequency spacing in the regions of compression (McDermott, 2011). FC is characterized by a cutoff frequency (CF) and by a compression ratio (CR), with lower cutoff frequencies and higher compression ratios representing more aggressive processing and greater amounts of signal distortion.

### Frequency Compression and Working Memory Capacity: Empirical Findings

Using a hearing-aid simulation of FC based on sinusoidal modeling, Arehart et al. (2013) considered the relationship between working memory and the combined effects of distortion caused by noise and FC in a group of older listeners with hearing loss. Results showed that age, hearing loss and working memory were all significant factors associated with degraded ability of listeners to process noisy speech processed with FC. Listeners with greater hearing loss, poorer working memory and more advanced age had the lowest intelligibility of frequency-compressed noisy speech. A follow-up study (Souza et al., 2015) found similar effects when FC was combined with wide-dynamic range compression. Similarly, in a neural network model of listener response to FC, Kates et al. (2013) showed that working memory was an important factor in perceptual response to FC for listeners with greater degrees of hearing loss but not for listeners with more mild hearing losses.

Ellis and Munro (2015) studied the relationship between FC and working memory capacity in a small group of older adults with moderate-to-severe high-frequency hearing loss. Because participants were part of a clinical trial with wearable (commercial) hearing aids, they had time to acclimatize to the FC processing. Listeners had customized FC parameters based on their hearing loss. Greater high-frequency hearing loss was positively correlated with FC benefit, but cognitive measures were not.

### Summary

As with NR processing, the relationship between working memory and response to FC processing is mixed (**Table 3**) and may be due to a number of different factors. For example, the specific implementation of FC differed in the studies of Arehart and colleagues compared to work conducted by Ellis and Munro. The experimental approach differed between the two research groups. The Arehart group used a simulated hearing aid such that effects of noise and FC were controlled, such that all listeners with hearing loss got the same amounts of FC processing. This had the advantage of being able to consider the relationships of working memory capacity and hearing loss and the cumulative effects of signal degradation caused by both noise and signal processing but also lacked using wearable hearing aids in clinical fittings. In contrast, Ellis and Munro (2015) used commercially available hearing aids and customized the amount of FC based on the individual listener’s audiogram. While having strong clinical face validity, their listeners also received different amounts of actual signal processing. Such differences may have contributed to differences across studies, and may also speak to the importance of individual customization in achieving best outcomes.

## Conclusion, Future Directions, And Clinical Applications

A growing body of work suggests that individuals with smaller working memory capacity may be more susceptible to an altered acoustic signal, such as might be produced by various types of hearing aid processing. The evidence is strongest for fast-acting WDRC, where nine studies have shown similar patterns. In each case, listeners with smaller working memory capacity (as measured with a reading span task) performed better with slow-acting than with fast-acting WDRC. One study (Cox and Xu, 2010) showed a relationship between working memory and compression speed, but in the opposite direction. Concerning FC, evidence for a relationship with working memory is mixed. Two studies by the same group using hearing aid simulations showed a relationship; a different study using wearable aids with customized hearing-aid parameters did not. Concerning NR processing, evidence for a relationship with working memory is weakest, with those few studies that observed a relationship producing incongruent outcomes. In this context, it should be noted that the signal processing conditions and outcome measures were rather dissimilar across the studies on NR effects.

To resolve the apparent discrepancy concerning the relationship between working memory and different types of hearing aid processing it would be useful to conduct research to assess the response to a number of hearing aid processing conditions (e.g., WDRC vs. NR) within the same group of individuals using the same outcome measures (e.g., speech understanding or memory performance). In this manner, it would be possible to find out whether the influence of working memory capacity on WDRC outcome generally translates to the domain of NR processing or not. Along those lines, it would also be useful to compare different types of NR processing (e.g., binary mask- vs. binaural coherence-based NR) within the same group of individuals. In this manner, it would be possible to assess the influence of specific NR design choices on the effects of working memory capacity. A more complete understanding of the role of working memory on speech understanding in listeners wearing hearing aids may also require consideration of how the signal alterations caused by a single type of signal processing interact with other concurrently implemented processing algorithms. In addition, in the context of the ELU model it may be important to consider how the cumulative effects of degradation caused by signal processing interact with other forms of signal degradation including the degree of hearing loss and the amount and type of noise in the environment. Irrespective of the actual research question, it would be important to characterize the effects of the signal processing conditions under consideration objectively (e.g., in terms of SNR improvement or amount of speech distortion). In this manner, it would be possible to rule out (or identify) factors (or confounds) that co-vary with working memory.

Given the aforementioned relationship between the strength of association between WDRC and working memory and acclimatization, longitudinal investigations would be beneficial to gain a better understanding of any long-term effects. For instance, it is possible that individuals with smaller working memory capacity who initially are disadvantaged by fast-acting WDRC in the long run would benefit from the greater audibility that it provides relative to slow-acting WDRC. It would also be important to extend this line of research to the domain of FC, and perhaps also to digital NR (although in this case continuous exposure would not be possible).

The role that working memory measurements may play in clinical care is an emerging issue. In contrast to laboratory studies, many of which focused on speech recognition, hearing aid benefit is multidimensional. For example, studies to date have noted a relationship between working memory and objective speech recognition, but also between working memory and the subjective benefits of different processing in the listener’s own environment. For the assessment of working memory to be feasible in the clinic, tasks are needed that can be administered within a reasonable amount of time (e.g., 5 min), that are acceptable for both the audiologist and the client, and for which scores can be quickly obtained and easily interpreted. The reading span task that has widely been used in the research studies summarized above is rather strenuous and typically takes around 15 min to complete (with 54 test items). A shorter version has been developed (e.g., Ng et al., 2013), but is not widely used. It may also be useful to consider measures of working memory capacity that bear close resemblance to the problems encountered by typical hearing aid candidates (i.e., that are more life-like); or, alternatively, components of working memory that lend themselves more readily to time-efficient testing in a clinical environment.

Clinical audiologists have shown great interest in the relationships between working memory and hearing aid benefit. Over the past few years, many clinical conferences have featured keynote speakers who work in the areas of cognition, hearing and aging. Clinicians have indicated a willingness to incorporate cognitive measures provided they offer improved hearing aid outcomes and/or better patient care. In addition to the need for time-efficient tests of working memory, the current review has identified several issues needing clarification. Given some of the uncertainties, such as the unclear role of contextual information, more controlled studies are needed to define the boundaries of the working memory-hearing aid effects, so that these relationships can be capitalized on to enhance hearing care.

## Conflict of Interest Statement

The authors declare that the research was conducted in the absence of any commercial or financial relationships that could be construed as a potential conflict of interest.

## Footnotes

1 The ratio of increase in input level to increase in output level.

2 The attack time is the time for the compressor to activate and stabilize (i.e., reach maximum compression) as input level increases; the release time is the time for the compressor to deactivate and stabilize (i.e., return to linear gain) as input level decreases.
